# Physical and psychosocial quality of life in children with overweight and obesity from Sri Lanka

**DOI:** 10.1186/s12889-020-10104-w

**Published:** 2021-01-07

**Authors:** S. Gunawardana, C. B. Gunasinghe, M. S. Harshani, S. N. Seneviratne

**Affiliations:** 1 Faculty of Medicine, University of Colombo, No 25, Kynsey road, Colombo, Sri Lanka; 2 Department of Pediatrics, Faculty of Medicine, University of Colombo, No.25, Kynsey Road, Colombo, Sri Lanka

**Keywords:** Childhood obesity, Quality of life, PedsQL, South Asia

## Abstract

**Background:**

While childhood obesity is rising rapidly in South Asia, there is limited research on quality of life (QoL) of children with overweight and obesity from the region. This study assessed physical and psychosocial QoL in Sri Lankan children attending a specialized obesity clinic, from both children’s and parents’ perspective, and modifiable social factors affecting QoL.

**Methods:**

We performed cross-cultural translation of the Pediatric Quality of Life Inventory (PedsQL™) 4.0 (Child-Self Report and Parent-Proxy forms), and assessed self-reported and parental-perception of physical and psychosocial QoL in 8–12 year-olds with overweight and obesity (*n*=110), referred for obesity management at a tertiary-care children’s hospital in Sri-Lanka. Body mass index (BMI) and pre-selected social factors affecting QoL were also assessed. Data were analyzed by non-parametric tests (Mann-Whitney U test, Wilcoxon test and Spearman correlation).

**Results:**

The median physical QoL was lower than psychosocial QoL (78.1vs81.7, *p*=0.032) and physical QoL was inversely correlated with BMI. Parental-perception of children’s physical and psychosocial QoL correlated with child-reported QoL, but was lower. Being bullied (*p*=0.001) and not getting regular exercise (*p*=0.031) were associated with lower psychosocial QoL. Both physical and psychosocial QoL were lower in children having difficulties in finding suitable clothes (*p*< 0.001).

**Conclusions:**

Children with overweight and obesity from Sri Lanka appeared to have greater impairment of physical QoL than psychosocial QoL. Higher BMI, bullying, lack of regular exercise and lack of suitable clothing, negatively affected QoL. Potential strategies to improve QoL include promoting regular exercise, addressing bullying and promoting availability of children’s clothes in larger sizes to fit children with overweight and obesity.

## Background

Childhood overweight and obesity is increasing rapidly in Sri Lanka. In 2016, the global prevalence of childhood obesity was 7.8% in boys and 5.6% in girls [1, 2]. A recent survey among 5–18 year olds in urban Sri Lanka showed an obesity prevalence of 10.3% and overweight prevalence of 11.3% [3]. Obesity has a negative impact on all aspects of child and adolescent health, including their physical, psychological and social well-being. Lifestyle factors and metabolic complications associated with childhood obesity have been studied previously in Sri Lankan children [4–6], but there is a paucity of research on impact on their quality of life including physical and psychosocial well-being.

Quality of life (QoL) describes the subjective wellbeing of an individual centered on his/her perception of life, based-on expectations, goals and standards [7]. QoL is a multidimensional variable including physical, psychological and social aspects. Both generic and disease-specific tools are available to assess health-related QoL [8]. Generic tools are especially useful for making comparisons between healthy and unhealthy individuals [8]. Assessment of QoL differs between children and adults, due to differing influence from family, school, and friends, and reduced ability of children to make significant changes to their context by themselves [9]. Thus special generic tools have been developed to measure QoL of children such as the Pediatric Quality of Life Inventory (PedsQL) and Child Health Questionnaire (CHQ), which assess children’s quality of life from their own as well as their parents’ perspective [8, 10].

Previous studies from high income countries have shown that QoL of children with overweight and obesity are lower than children of normal weight, and that QoL deteriorates with increasing body mass index [11, 12]. Indeed, one study revealed that QoL of severely obese children was comparable to children suffering from cancer [13]. However, despite childhood obesity being a rising problem in South Asia, there is a lack of studies on QoL from the region. Further, there are considerable socio-cultural differences such as high parental expectations and different societal perceptions on ideal body weight and lifestyle standards [14]. Thus QoL data from other regions cannot be directly extrapolated to South Asian children. Furthermore, previous researchers have highlighted the need of exploring potentially modifiable factors affecting QoL of children with overweight and obesity, to identify and implement strategies to improve QoL [11, 15]. Therefore, we conducted this study to assess physical and psychosocial QoL in Sri Lankan children with overweight and obesity aged 8–12 years using a generic international QoL questionnaire (PedsQL™ 4.0) and explore social factors including potentially modifiable factors associated with lower QoL. We hypothesized that physical and psychosocial QoL would decline with increasing BMI, and be lower in obese compared to overweight children.

## Methods

### Study design and setting

This cross-sectional, observational study was conducted between July to August 2016 at the Professorial Unit specialized obesity and nutrition clinic of Lady Ridgeway Hospital, Colombo, the largest and main tertiary care pediatric referral center in Sri Lanka. Ethical clearance was obtained from Ethics Review Committee of the Faculty of Medicine, University of Colombo and Lady Ridgeway Hospital. Information sheets explaining purpose, risks and benefits of the study were provided, and informed written consent obtained from all participating families.

### Study population

The study sample consisted of 110 children aged between 8 and 12-years with a body mass index (BMI) >+1SD, presenting to the obesity/nutrition clinic during the study period. Exclusion criteria included major disability, chronic medical complications and receiving obesity-related interventions > 1 year.

### Data collection and measurements

#### Quality of life

Child Self-Report and Parent Proxy-Report forms of the PedsQL™ 4.0 Generic Core Scales (8–12 years) [10], a validated international questionnaire [16, 17] was used to assess QoL. Original English questionnaires were cross-culturally translated into Sinhalese as per standard linguistic validation guidelines. Forward translation was done by two independent translators and a single reconciled Sinhalese version was produced which was back-translated into English and compared with the source instrument to make amendments to the forward translated version. Pilot testing was done using the respondent debriefing method, and confirmation of appropriate linguistic validation was obtained for the final translated questionnaire.

The PedsQL questionnaire for 8–12 year old children is a self-administered questionnaire which contains 23-items classified under four subscales (physical, emotional, social, and school functioning scales). For each item, 5 options were provided: never a problem; almost never a problem; sometimes a problem; often a problem; or almost always a problem, and 0–4 marks allocated accordingly. Appropriate items were reverse scored and linearly transformed to a 0–100 scale. Higher scores indicate better QoL. Physical QoL was derived from physical functioning subscale, psychosocial QoL was derived from emotional, social and school functioning subscales and overall QoL was derived from all four subscales [10].

#### Socio-demographic data and factors associated with QoL

An additional questionnaire was used to collect socio-demographic data (age of child, gender, district of residence, religion, ethnicity) and possible/perceived modifiable social factors influencing QoL of children with overweight/obesity (being bullied by other children, having peers (siblings/close friends) with similar body habitus, being on diet control, obtaining adequate exercise, facing difficulties in finding suitable clothes that fit). Social factors were based on information gathered during prior group-counseling sessions (child-focused family-based round-table discussions for 60–90 min) conducted on random clinic days for children with overweight/obesity (BMI>+1SD for age [WHO standards]) at the clinic, by the last author (SNS) in the year proceeding this study.

The translated, self-administered PedsQL™ Child Self-Report questionnaire was provided to each child and the self-administered Parent Proxy-Report to the accompanying parent. Parents and children were seated separately, to facilitate children’s independency. An investigator was present to clarify any doubts. The additional questionnaire was filled in collectively by parent and child.

#### Anthropometric measurements

Height of children was measured using a stadiometer (Seca 213 portable stadiometer, Seca GmbH & Co.KG, Germany) and weight using a weighing scale (Seca 803 digital flat scale, Seca GmbH & Co.KG, Germany) complying with standard techniques. BMI was calculated using the standard equation; dividing weight in kilograms (kg) by height in meters squared (m^2^). World health organization (WHO) BMI z-score charts for age for girls and boys were used to categorize children as overweight and obese (BMI>+1SD=Overweight, BMI>+2SD=Obese, BMI>+3SD=Severely Obese) [18].

### Data analysis

As QoL data showed non-normal distribution, central tendency was described using median and inter quartile range (IQR), and analyzed by appropriate non-parametric tests, using SPSS statistical software. Child-reported and Parent-reported QoL scores were compared using Wilcoxon test, while associations between the two were assessed by Spearman correlation. Child-reported QoL was used for analysis of associations with BMI and social factors. Comparison between overweight (BMI >+1SD to +2SD) and obese children (BMI> +2SD), and influence of social factors were analyzed by the Mann-Whitney U test while associations with BMI were assessed by Spearman correlation. Significance level was set at 0.05 for all tests. Effect size of correlations was evaluated by Cohen’s standards (correlation coefficients of 0.10–0.29, 0.30–0.49 and ≥0.50 representing small, medium and large associations respectively) [19].

## Results

### Socio-demographic characteristics

One hundred and ten children participated in the study. Average BMI was 24.15± 2.85 kg/m^2^.

Majority of the participating children were obese 77 (70%) with 8 (7%) being severely obese, while 33 (30%) were overweight. Table 1 describes the basic socio-demographic characteristics of participating children. There was a male preponderance (63% male vs 37% female). Participants from all main ethnicities and religions in Sri Lanka were included.

**Table 1:** General Characteristics of the Study Population

Variable	Category	All participants (*n*=110) number = n	Percentage%
Age (years)	8 - < 10 years	66%	60%
	10 - < 13 years	44%	40%
Gender	Male	69	62.7%
	Female	41	37.3%
Ethnicity	Sinhala	90	81.8%
	Tamil	8	7.3%
	Muslim	12	10.9%
Religion	Buddhist	82	74.5%
	Catholic	11	10.0%
	Islam	12	10.9%
	Hindu	5	4.5%
District of residence	Colombo district	62	56.4%
	Other districts	44	40.0%
	No response	4	3.6%
BMI category	Overweight	33	30.0%
	Obese	69	62.7%
	Severely Obese	8	7.3%

### Child self-report and parent-proxy-report QoL

Median [IQR (Inter Quartile range)] Child Self-Report physical QoL, psychosocial QoL and overall QoL were 78.1 [65.6, 88.4], 81.7 [72.5, 90.8] and 80.4 [69.6, 89.5], respectively. The median physical QoL was lower than psychosocial QoL (78.1 vs 81.7, *p*=0.032).

The median [IQR] physical QoL, psychosocial QoL and overall QoL as reported by parents were 75.0 [56.3, 87.5], 76.7 [65.0, 86.7] and 74.5 [60.9, 87.0] respectively. Comparisons and correlations between *Child Self-Report* QoL and *Parent-Proxy Report* QoL scores are shown in Table 2. Children’s Self Report QoL scores were higher than parental perception scores. However, there was medium to large positive correlation between children’s self reported and parents’ perception of QoL.

**Table 2:** Comparison and Correlation between Child Self-Reported and Parent Proxy-Reported QoL

	*PedsQL ChildSelf-Report Scores* median (IQR)	*PedsQL Parent-Proxy-Report Score* median (IQR)	*p*-value^a^	Spearman’scorrelation coefficient (r)^b^	Significance of correlation coefficient^b^
Physical QoL	78.1 (65.6, 88.4)	75.0 (56.3, 87.5)	0.005**	0.399 (medium)	< 0.01**
Psychosocial QoL	81.7 (72.5, 90.8)	76.7 (65.0, 86.7)	0.017*	0.442 (medium)	< 0.01**
Overall QoL	80.4 (69.6, 89.5)	74.5 (60.9, 87.0)	0.001**	0.515 (large)	< 0.01**

^a^Significance of difference between *PedsQL Parent Proxy-Report Scores and PedsQL Child Self-Report Scores*

^b^Correlation between *PedsQL Parent- Proxy-Report Scores and PedsQL Child Self-Report Scores.* Effect size was evaluated using Cohen’s standards: with correlation coefficients of 0.10-0.29, 0.30-0.49 and ≥0.50 representing small, medium and large associations respectively [19].

*Statistically significant at p<0.05 level **Statistically significant at p<0.01 level

*QoL, Quality of Life; IQR, Inter Quartile Range*

### Association between PedsQL child self-report scores and BMI

As hypothesized, BMI showed small yet significant inverse correlation with physical QoL, as well as tendency for significant inverse correlation with psychosocial and overall QoL (Table 3). Considering psychosocial subscales, BMI showed significant negative correlation with social functioning, but not with emotional or school functioning (Table 3).

**Table 3:** Association of Child Self-Reported QoL (median [IQR]) in the total study population with BMI

	n	Median (IQR)^a^ (n=110)	*Spearman’s correlation coefficient (r*^b^*)*	Significance of correlation coefficient^b^
Physical QoL	109	78.1 (65.6, 88.4)	−0.197 (small)	0.040*
Psychosocial QoL	109	81.7 (72.5, 90.8)	−.0.164 (small)	0.088
Emotional functioning	110	85.0 (65.0, 91.3)	−0.083	0.391
Social functioning	109	85.0 (70.0, 95.0)	−0.237 (small)	0.013*
School functioning	109	85.0 (70.0, 90.0)	−0.081	0.400
Overall QoL	109	80.4 (69.6, 89.5)	−0.184 (small)	0.055

^a^Child Self-Report PedsQoL score

^b^Correlation between BMI values with QoL scores. Effect size was evaluated using Cohen’s standards: with correlation coefficients of 0.10-0.29, 0.30-0.49 and ≥0.50 representing small, medium and large associations respectively [19]

*Statistically significant at *p*<0.05 level

*IQR* Interquartile range, *QoL* Quality of Life, *BMI* Body Mass Index

When comparing BMI groups, overall QoL was lower in obese than overweight children (78.3 [69.0, 87.0] vs 83.7 [71.7, 93.5], *p*=0.049). Table 4 shows the comparison of physical and psychosocial QoL between overweight and obese groups. Compared to overweight children, obese children reported significantly lower physical QoL (75.0 [62.5, 87.5] vs 84.4 [71.9, 93.8], *p*= 0.028) while psychosocial QoL was not significantly lower (80.0 [71.7, 89.2] vs 83.3 [76.7, 93.3], *p*=0.106) (Table 4).

**Table 4:** Child Self-Reported Physical and Psychosocial QoL based on potentially modifiable factors (median [IQR])

Variable		n	Physical QoL^a^	*p*-value	Psychosocial QoL^a^	*p*-value
Degree of Obesity	Overweight	33	84.4 (71.9, 93.8)	0.028^*^	83.3 (76.7, 93.3)	0.106
	Obese	77	75.0 (62.5, 87.5)		80.0 (71.7, 89.2)	
Subject to bullying	Yes	25	75.0 (65.6, 84.4)	0.273	73.3 (60.0, 83.3)	0.001**
	No	83	81.3 (65.6, 93.3)		83.3 (75.0, 91.7)	
Regular exercise	Yes	67	81.3 (65.6, 93.3)	0.078	83.3 (75.0, 92.5)	0.031*
	No	42	75.0 (62.5, 84.4)		77.5 (61.7, 87.5)	
Difficulty finding fitting age-appropriate clothes	Yes	52	71.9 (57.8, 84.4)	0.002**	76.7 (60.0, 83.3)	0.001**
	No	56	84.4 (71.9, 93.8)		86.7 (77.5, 93.1)	

^a^Data are median (IQR). *Statistically significant at *p* <0.05 level. **Statistically significant at *p*<0.01 level

*QoL* Quality of life, *IQR* Inter Quartile Range

### Child self-reported physical and psychosocial QoL based on potentially modifiable factors (median [IQR])

Child Self-Reported physical QoL and psychosocial QoL based on modifiable factors are shown in Table 4. Physical QoL was lower in children who had difficulty finding appropriate clothing, and showed tendency to be lower in those not engaging in regular exercise. Psychosocial QoL was highly significantly lower in children subject to bullying and those having difficulty in finding appropriate clothes, and significantly lower in those not engaging in regular exercise. Both Physical and Psychosocial QoL did not vary significantly based on gender, presence of siblings or close friends with overweight/obesity or being on diet control.

## Discussion

### Summary of findings

Sri Lankan children with overweight and obesity in this study had lower physical QoL (median 78.1 [65.6, 88.4]) than psychosocial QoL (median 81.7 [72.5, 90.8]). As hypothesized, obese children had greater impairment of overall and physical QoL compared to overweight children, and increasing BMI had a negative effect on physical QoL. On the contrary, psychosocial QoL did not show significant correlation with increasing BMI except in the social functioning subscale, and showed stronger associations with modifiable social factors, such as bullying, having difficulty finding appropriate clothes, and lack of regular exercise Parental perception of QoL showed good correlation with, but was lower than, children’s self-reported physical and psychosocial QoL.

### Comparison with previous research

#### Comparison with QoL of children with overweight/obesity from other regions

Sri Lankan children with overweight/obesity appeared to have similar physical QoL compared to children with overweight/obesity elsewhere in the world, with other studies reporting mean/median PedsQL™ physical QoL ranging from 67.2 to 88.4 [13, 20–25]. Our study population however, appeared to have higher psychosocial QoL compared to mean/median psychosocial QoL from other studies which generally ranged from 62.5 to 79.9. Further, most other studies reported lower psychosocial QoL, compared to physical QoL, which differed in our study [13, 20–22, 24, 25].. Therefore, Sri Lankan children with overweight and obesity appear to have equally compromised physical QoL compared to similar children from other regions of the world, but they appear to show better preservation of their psychosocial QoL. Cultural attitudes towards childhood obesity in the Sri Lankan population could have played a role in the comparatively higher psychosocial QoL in Sri Lankan children with overweight and obesity. In Sri Lanka, being overweight and obese is not considered as an illness, and many parents prefer their children to be plump rather than thin [26]. Further, increased weight/BMI is sometimes considered as an indicator of strength, attractiveness and even prosperity in children and adolescents, whereas thin children, are considered to be weak [26]. Thus, children with overweight and obesity might be less psychologically and socially disadvantaged in contrast to the cultural context in western countries.

With regard to parental perception of QoL, many studies mirrored our findings, demonstrating that parents perceived lower QoL compared to that self-reported by children with overweight and obesity, albeit with good positive correlation between the two [11, 12, 23]. One explanation suggested is that parents could be more conscious of long-term negative consequences of obesity in their children, while children may have a more short-term view, and thus a more positive outlook [27].

#### Comparison with QoL of children with other conditions

When considering PedsQL physical QoL described by Varni et al., Sri Lankan children with overweight and obesity appeared to have lower physical QoL than healthy children (84.4), more comparable to QoL of acutely ill children of similar age in United States (78.9). However, psychosocial QoL score appeared more comparable to healthy children (82.4), than acutely and chronically ill children (78.7 and 77.1 respectively) [16]. Closer to home, a Sri Lankan study which assessed QoL using PedsQL™ in children with asthma and healthy controls aged 12–14 years, reported mean physical QoL of 88.7 and psychosocial QoL of 86.3 in healthy control group children, and physical QoL of 78.8 in children with asthma [28], which appeared higher than the scores we obtained for Sri Lankan children with overweight and obesity, although children with asthma had lower psychosocial QoL (74.9) [28]. Thus Sri Lankan children with overweight and obesity appeared to have lower physical QoL than similar aged healthy children, which was lower /similar to that of children with asthma and acute illnesses, while their psychosocial QoL was more comparable to healthy children and better than children with acute/chronic illness based on average PedsQL scores.

While childhood obesity is sometimes considered favorably socio-culturally in Sri Lanka and other South Asian countries, asthma is socio-culturally considered a more serious and debilitating chronic illness by South Asians [29]. This could perhaps explain why Sri Lankan children with asthma have lower psychosocial QoL, while psychosocial QoL of Sri Lankan children with overweight and obesity remain more comparable to healthy children. Physical QoL in Sri-Lankan children with overweight and obesity, where socio-cultural influences will not confer a similar protection, however, is lower than in healthy children, and more comparable to that of acutely ill children reflecting the extent of difficulties in physical activities and physical discomfort faced by these children due to obesity.

#### Factors associated with QoL

When considering the relationship between QoL and BMI, a decrease in QoL with increasing degree of obesity, as seen in this study, has been described by previous studies [22, 23]. Further, as seen in our study, several previous studies have reported a strong to moderate negative relationship between degree of obesity and QoL in physical and social functioning, with emotional and school functioning remaining relatively less affected [12, 22, 23, 30]. These results suggest the relative resilience of children with overweight and obesity, to preserve their school functioning and emotional wellbeing in the context of increasing severity of obesity, irrespective of geographical or cultural background. Similarly, decrease in physical QoL noted with increasing BMI also appears to be a universal problem, irrespective of socio-cultural millieu. Further, while psychosocial QoL in Sri Lankan children with overweight/obesity appears to be higher compared children from western countries, both groups experience a decline in their social functioning with increasing severity of obesity.

Studies looking at QoL in relation to gender have shown controversial results, with some studies reporting a higher QoL in boys than girls [11, 12] and others reporting no difference [13, 25]. Our study findings collude with the latter. The complex and multidimensional nature of interaction between gender and obesity related QoL have been reported, and could explain these discrepancies [31].

There is limited published data on the association of social/modifiable factors with QoL of children with overweight/obesity. Previous studies have shown peer-victimization/ bullying to be associated with lower QoL in children overweight/obesity, which was mirrored by our study [11, 32]. Further we found a positive association between getting regular exercise and better psychosocial QoL, which corresponds with a previous study reporting lower psychosocial QoL in overweight children who are less physically-active [33]. Our study findings thus contribute valuable information on modifiable social factors associated with lower QoL in children with overweight/obesity to the global literature.

### Implications for clinical practice

Research on social factors associated with impaired QoL in children with overweight/obesity is important to gain a deeper insight into the issue, and to identify new approaches to improve QoL [11, 15]. In this study, we studied associations of QoL in children with overweight/obesity with some novel social factors such as: difficulty in finding/ fitting in to age-appropriate clothes; being on diet control; and having siblings or friends with overweight/obesity; which have not been reported before. Interestingly, we found both physical and psychosocial QoL to be lower in children with overweight and obesity who had difficulties in finding suitable clothes. This finding is further supported by a previous study showing that buying clothes was an activity that obese children disliked [34]. This is a less well recognized factor that, perhaps, could be addressed by highlighting this issue to stakeholders in the clothing industry in the locality, to help ally a common problem faced by children with overweight/obesity in everyday life, which is impairing their QoL.

The strong association of bullying with lower psychosocial QoL of children with overweight and obesity demonstrated in this study, reinforces findings of several previous studies, and highlights the urgent need for better measures to reduce obesity-related bullying to improve the wellbeing of these children. Better classroom support, encouraging peers to give better support, and increasing coping skills of children with overweight and obesity could be helpful strategies [34–36]. Importance of engaging in regular physical activity/exercise, and its positive associations with psychosocial QoL of children with overweight and obesity has been demonstrated in this study, and as well as previously [33]. Therefore, promoting exercise among children with overweight/obesity appears important, not only for weight management and metabolic health, but also for improving psychosocial wellbeing.

Further, given the findings of this study, on relative preservation of psychosocial QoL especially in emotional and school functioning subscales in Sri Lankan children with overweight and obesity, future research and programs for management of childhood obesity, could try to understand and enhance the resilience of these children in emotional and school functioning, in addition to focusing on improving physical and social wellbeing in these children [14, 23].

#### Strengths and limitations

Strengths of this study include using a pre-validated, international generic QoL questionnaire, adhering strictly to standardized translation procedures as outlined, which increased validity and enabled comparison with similar QoL data from children from other regions and with other conditions, to obtain a more in-depth appreciation of the impact of overweight/ obesity on QoL.

Limitations of this study are the relatively small study sample, lack of a control group, and the study being conducted in a clinical rather than a community setting. Inclusion of a control group of healthy children would have provided useful information. Availability of a cross-culturally adapted PedsQL questionnaire will be helpful for the conduct of future studies in this area. However, while the questionnaire was translated according to standard protocol with cross cultural adaptation, and certified as linguistically validated, psychometric validation was not conducted. Reliability of obtained results would increase if the questionnaire was psychometrically validated. With regard to the study setting, a few previous studies have compared QoL in children with overweight/obesity between community and clinical settings, where some studied have shown no difference in QoL scores in hospital-based versus community settings [23], while others have shown lower QoL in clinical settings [11]. Conversely, the study setting also helped to increase the generalisability of the results. As this study was conducted at a tertiary care referral centre, 40% of participants were referred from districts other than Colombo district. Our study population thus included families living throughout the country as well as representing all main ethnic and religious groups in Sri Lanka. Thus, we believe that the study findings are generally applicable to children with overweight/obesity in Sri Lanka.

## Conclusions

To the best of our knowledge, this is the first study to assess QoL in Sri Lankan children with overweight and obesity. Our findings emphasize the importance of addressing impairment of QoL faced by Sri Lankan children with overweight and obesity, especially in physical functioning where most distress was documented. Further, parental perceptions were lower, but showed good correlation with child self-reported QoL, which signifies that parents are quite attuned to their children’s issues, and possibly more concerned about impaired wellbeing of their child, than the children themselves. This emphasizes the importance and value of parental involvement in management of childhood obesity.

This study documents for the first time, novel modifiable social factors associated with impaired QoL of children with overweight/obesity in Sri Lanka, such as difficulties in finding age-appropriate clothes that fit, as well as previously documented factors such as bullying. It also demonstrates for the first time, the protective effects of regular exercise on psychosocial QoL of Sri-Lankan children with overweight/obesity and the relative resilience of these children in emotional and school functioning, even in the context of increasing BMI. Prevention of peer victimization of children with overweight/obesity, promoting physical activity and enlightening the clothing industry on the need of a range of children’s clothes to fit larger children, are potential strategies which could improve QoL of children with overweight/obesity in Sri Lanka, as well as elsewhere.

## Data Availability

The datasets used and/or analyzed during the current study are available from the corresponding author on reasonable request.

## Abbreviations

QoL: Quality of Life
PedsQL: Pediatric Quality of Life inventory
BMI: Body Mass Index
WHO: World Health Organization
IQR: Inter Quartile Range.
